# Online/Cyber Counseling Services in the COVID-19 Outbreak: Are They Really New?

**DOI:** 10.1177/1542305020948170

**Published:** 2020-09-23

**Authors:** Dominikus David Biondi Situmorang

**Affiliations:** Department of Guidance and Counseling, Atma Jaya Catholic University of Indonesia, Indonesia

**Keywords:** Online, cyber, counseling, COVID-19, outbreak

## Abstract

Online/cyber counseling has been named as the best way to offer counseling services during the COVID-19 outbreak. The purpose of this article is to explore the use of online/cyber counseling during the COVID-19 outbreak to solve psychological problems. The author examines the history and concepts, the therapeutic relationship, transference and countertransference, the advantages along with the disadvantages, considerations, implications, and curriculum for online/cyber counseling during COVID-19 outbreak.

## Introduction

Online/cyber counseling has been characterized as the conveyance of counseling services via the Internet, where the pastoral/spiritual counselor or psychologist and counselee/client are not within the same physical area and they communicate utilizing computer-mediated communication innovations (Abney & Cleborne, 2004; Baker & Ray, 2011; Richards & Viganó, 2012). An assortment of modalities has been recognized as online/cyber counseling, including but not restricted to instant messaging, synchronous chat, text messaging, video-conferencing, and asynchronous email (Barak, Hen, et al., 2009; Barnett, 2005; Dowling & Rickwood, 2013).

Over the past two decades, online/cyber counseling has received much consideration and acknowledgment as a reasonable counseling conveyance strategy (Cohen & Kerr, 1998). Pastoral/spiritual counselors or psychologists who have adequate technical ability can progressively communicate with their counselee/client through the online medium and/or undertake the whole progress online (Mallen & Vogel, 2005). The benefits of online/cyber counseling incorporate more noteworthy availability, reaching populaces that would not immediately look for face-to-face counseling, reasonableness, and ease of record keeping (Leibert et al., 2006; McCrickard & Butler, 2005; Rochlen et al., 2004). Moreover, Suler (2002) and Partala (2011) expressed that counselees/clients taking part in online/cyber counseling are less likely to feel powerless for revealing their individual data and also feel less ashamed about their issues, due to the anonymity related to online/cyber counseling.

In any case, pundits of online/cyber counseling have raised concerns with respect to the reducing of visual prompts, failure to intercede in an emergency, and need of restorative control (Leibert et al., 2006; Richards, 2009; Rochlen et al., 2004). Numerous experts within the field have expressed concerns regarding moral issues of online/cyber counseling, including things such as competence, informed consent, privacy, and security (Barnett, 2005; McAdams & Wyatt, 2010; National Board for Certified Counselors, 2012). In addition, online/cyber counseling cannot be utilized by those who do not have get to satisfactory innovation or do not have the essential innovation aptitudes (West & Hanley, 2006).

In spite of the concerns around online/cyber counseling, an increasing number of analysts have found proof supporting the viability of online/cyber counseling for an assortment of mental issues, including depression, panic disorder, social anxiety, post-traumatic stress disorder, and eating disorders (Skinner & Zack, 2004; Sloan et al., 2011; Stamm, 1998). In addition, some research on counselee/client online counseling has uncovered empowering results. For instance, Cohen and Kerr (1998), Richards and Viganó (2012), and Rochlen et al. (2004) found that counselees/clients show the level of session profundity, fulfilment, and smoothness to be comparable between online/cyber counseling and face-to-face counseling. Furthermore, McKenna and Bargh (2000) and Reynolds et al. (2006) found that counselees/clients who experienced uneasiness and social separation were more likely to create deep connections through online/cyber counseling than through in-person counseling.

Research has shown that counselees/clients have demonstrated both benefits and progress related to online/cyber counseling services. Joinson et al. (2008) and King et al. (2006) subjectively investigated adolescents’ encounters with a 24-hour emergency counseling service through text-messaging. The participants projected a need for individual contact, anonymity, and the capacity to re-read and alter their written explanations as positive aspects of online/cyber counseling administrations, in spite of the fact that their main concerns were about how their pastoral/spiritual counselors or psychologists would be able to explore their feelings and sentiments through a texting format. Kanani and Regehr (2003) and Young (2005) conducted an overview of those who had shown an interest in online chat sessions for compulsion issues and found that members declared that their fundamental reasons in looking for online/cyber counseling are secrecy, comfort, and the pastoral/spiritual counselor’s or psychologist’s qualifications (e.g., considerable experience and academic qualifications). Be that as it may, they also stated the concerns related to the need for visible security, and security related to online/cyber chat sessions (Dowling & Rickwood, 2013).

At the time of the COVID-19 pandemic outbreak, human activities were no longer the same as before. Various countries in the world imposed regulations for working from home, studying from home, social distancing, physical distancing, etc. Furthermore, these adjustments may eventually have triggered mental health symptoms, such as anxiety, depression, stress, and so on (Arnout et al., 2020; Liebrenz et al., 2020).

Regarding this phenomenon, mental health services can no longer be done face-to-face, because pastoral/spiritual counselors or psychologists and counselees/clients must conduct social and physical distancing during the counseling process, but psychological distancing is not permitted. This has ultimately led to all pastoral/spiritual counselors or psychologists around the world implementing an online/cyber counseling process, whether through chat, email, WhatsApp, video call, or even telephone. At present, during this COVID-19 outbreak, the best solution is the use of online/cyber counseling.

## The History and Concept of Online/Cyber Counseling

The history of online/cyber counseling began with the term “distance counseling”. Distance counseling has existed since the initiation of modern mental wellbeing. Freud and Morita connected with counselees/clients regarding the side effects and treatments (Barnett, 2005; France et al., 1995). Phones have been utilized for emergency lines since the 1950s and are at present broadly utilized for aide services (Allerman, 2002; Centore & Milacci, 2008; Mohr et al., 2008; Stead et al., 2013), and counseling by means of video has been utilized since the 1960s (Holmes & Foster, 2012; Rohland, 2001). For each of these, there is a modern computer-mediated equivalent. Advanced letter composition regularly happens through mail and electronic bulletin boards; synchronous connections (i.e., those taking part at the same time as the pastoral/spiritual counselor or psychologist and counselee/client) incorporate voice-over-Internet protocol (VOIP), video-conferencing apps, instant messaging (IM), and chat, which contrasts with short-message service (SMS), its phone-based counterpart (Centore & Milacci, 2008; Kessler et al., 2009; McCrickard & Butler, 2005; Mallen & Vogel, 2005).

The utilization of Internet services is on the increase and it is obvious that the preparation of student pastoral/spiritual counselors or psychologists to work within this medium must be advanced to take account of such social developments (Mishna et al., 2013). Over a long period, the use of online/cyber innovation to supply counseling sessions has been developed (Allerman, 2002; Baker & Ray, 2011; Pollock, 2006). A few accept with indiscreet eagerness (Hennigan & Goss, 2014; Kettunen et al., 2013; Prado & Meyer, 2004). In agreement with that, according to Glasheen and Campbell (2009) and King et al. (2006), online/cyber counseling has become a reasonable choice for helping young people when they are confronting mental wellbeing challenges. Counselee/client request for such services are anticipated to significantly increase in the long term (McKenna & Bargh, 2000; Norcross et al., 2002; Stamm, 1998; Wright, 2002) due to easy access to the Web.

With the progress that is happening in the cyber world it is recommended that there should be critical suggestions for improvement in supporting counseling offices such as: instruction, mental wellbeing, and social work (Kettunen et al., 2013). Kessler et al. (2009) and Mishna et al. (2013) accept that this will require more efficient consideration, mindfulness-raising, and discussion of online issues, and an introduction to the necessary aptitudes which will need to be learned. Also, this new information must be coordinated with preparation for counseling.

Research supports an assortment of terms used to depict online/cyber counseling, such as cyber counseling; online or Internet therapy; e-counseling; e-therapy; email therapy; Internet counseling and Web counseling, to name a few (Allerman, 2002; Cook & Doyle, 2002; Jones, 2013; Lau et al., 2013; McCrickard & Butler, 2005; Pollock, 2006). Also, online/cyber counseling can be conveyed through a variety of distinctive media, by means of phone (Mallen & Vogel, 2005; Mohr et al., 2008), non-concurrent mail, content chat or messages posted on discourse-board chat rooms (Joinson et al., 2008; Jones, 2013), genuine time synchronous chat through Web-based and face-to-face Webcam sessions (Bambling et al., 2008; Cook & Doyle, 2002). Richards (2009) and Rohland (2001) conclude that professionals cannot disregard the influence of such innovation, and Lau et al. (2013) and Wagner et al. (2014) recommend that at whatever point an Internet counselee/client is communicating in a virtual world, their problems should always be regarded as genuine.

According to Marton and Booth (1997, p. 111) and Rochlen et al. (2004), a person’s understanding and involvement of wonders (in this research about online/cyber counseling) are entwined with their capacity to act—since “you cannot act other than in connection to the world as you encounter it.” Kettunen et al. (2013) and Goss and Anthony (2004) support this conviction, which was originally hypothesized by Kember (1997) and Akerlind (2003, 2008); that a broader understanding of practitioners’ conceptions is to begin with required some time recently arranging preparing mediations. They conjecture that this will affect a person’s or a student’s capacity to adjust to unused advances in their skills (Situmorang & Salim, 2020).

## The Therapeutic Relationship in Online/Cyber Counseling

There is no proof that the relationships between counselee/client and pastoral/spiritual counselor or psychologist are exceptionally diverse when counseling treatments are given over the Internet. The foremost self-evident contrast is the need for non-verbal signals (Abney & Cleborne, 2004; Liess et al., 2008). Whereas this clearly unfavorably influences the strategies utilized in conventional counseling, a few have contended that such disadvantages are, at the least, mostly counterbalanced by the favorable characteristics of text-based communication (Suler, 2004). For example, a few have contended that counselees/clients unveil at a faster rate when making contact via email (Barnett, 2005; Rochlen et al., 2004), which online/cyber back bunches may have a ‘‘disinhibiting effect’’ (Allerman, 2002; Barak et al., 2008). Clearly, counselees/clients are more likely to get straight to the point instead of steadily easing into an articulation of the issue (Hennigan & Goss, 2014; Rochlen et al., 2004; Suler, 2004). This divulgence may be candidly noteworthy in spite of being text-based (Bar-Lev, 2008; Centore & Milacci, 2008).

The revelation of their problems may be increased by the sense of privacy that counselees/clients may experience by not being physically seen or by not having to disclose their character (Cartreine et al., 2010). Revelation may be advanced and encouraged by the orderly introduction of web-based methods (Joinson et al., 2008; Jones, 2013). Moreover, certainly requiring the counselee/client to verbalize their concerns in a written format may create a more intelligent position on the part of the counselee/client (Hennigan & Goss, 2014; Rochlen et al., 2004). Composed communication permits counselees/clients to consider what they are “actually saying” and to change their explanations without worrying that it will come across incorrectly the first time. This could be more than just the original communication made by a pastoral/spiritual counselor or psychologist.

## The Issue of Transference in Online/Cyber Counseling

Transference may be a central occasion which decides the relationship between the counselee/client and the pastoral/spiritual counselor or psychologist in each psychotherapeutic experience (Kanani & Regehr, 2003; McCluskey & O’Toole, 2019; Parth et al., 2017). In any case in which psychodynamic or classical psychoanalytic interventions are concerned, there is continuously an oblivious transference. Historically, this is often a disclosure of early psychoanalysis, which means in a smaller sense, the enthusiastic attitude of understanding a pastoral/spiritual counselor and psychologist has in the counseling process that initially offends childhood, but which is unconsciously expressed in the counseling process (Aggarwal, 2020; Greenberg, 2016). These incorporate, for example, early childhood-acquired love, desire, abhorrence, and neglect, which are connected within the treatment situation. It is an oblivious occasion, the realization and mindfulness of which may be at the center of psychodynamic and explanatory treatments (Aggarwal, 2020; McCluskey & O’Toole, 2019).

An ordinary transference in online/cyber counseling (especially during this COVID-19 outbreak), which may be connected with stress, anxiety, and depression in counselees/clients, regularly unfurls at the start of online/cyber counseling. The counselee/client feels frail and powerless, talks discreetly, and is unknowingly looking for maternal security—somebody who “gets” them and who can do everything for them. In the event that their unhappiness is related to early misfortune encountered in their childhood, as is frequently the case, their oblivious transference serves the craving to re-establish the circumstance some time to remind them of the painful events they have experienced (Parth et al., 2017; Veach et al., 2018). This may be related to idealizing trust, regarding the meaning of the transference perspectives to the pastoral/spiritual counselors or psychologists. In a more basic history, this transference situation is regularly found within the relationship with the counselee/client, whether female or male. The partner in the counseling process will unconsciously expect to get protection from us and we can become dominant figures for themselves.

## The Issue of Countertransference in Online/Cyber Counseling

Countertransference is the complementary response of the pastoral/spiritual counselor or psychologist, which ought, moreover, which should be explained in a broader sense, including all the enthusiastic responses given by the counselee/client (Aggarwal, 2020; McCluskey & O’Toole, 2019; Parth et al., 2017). That is, in a broader sense, countertransference is the full subliminal enthusiastic reaction of the individual giving treatment and a pastoral/spiritual counselor or psychologist group to the behavior of a counselee/client, which incorporates their responses and states of mind coming about from the transference (Gait & Halewood, 2019; Greenberg, 2016).

Within the pastoral/spiritual counselor or psychologist, sentiments of maternal and counselee/client-oriented assurance are activated. If this complementary response is not reflected, the pastoral/spiritual counselor or psychologist rapidly chooses a solid, supportive, and dynamic role. To begin with, the counselee/client unknowingly gives the pastoral/spiritual counselor or psychologist reassurance, by giving a slight indication of change. This, in turn, propagates the pastoral/spiritual counselor’s or psychologist’s behavior (Aggarwal, 2020; Gait & Halewood, 2019). When the involvement of torment and loss is at that point treated in online/cyber counseling, resistance becomes an issue; the counselee/client survives in a discouraged, pitiful state of mind, so as not to have to re-experience past trauma. As a resistance, which is additionally not realized, depressive indications are used against psychodynamic translation endeavors, which may take a long time. Because online/cyber counseling preparations are not functioning properly, transference problems arise within the pastoral/spiritual counselor or psychologist (Searles, 2017; Veach et al., 2018). Furthermore, Kehoe (2016), Pedhu (2019), Maximo (2019), and Peeters (2020) give detail information about countertransference and ethical considerations in spiritual/religious counseling and psychotherapy.

## The Advantages of Online/Cyber Counseling

There are a few advantages to online/cyber counseling. Counselees/clients and pastoral/spiritual counselors or psychologists do not have to meet each other within a physical space, subsequently decreasing the requirement for travel, appointment-related concerns, and overhead costs (Abney & Cleborne, 2004; Cartreine et al., 2010; Green-Hamann et al., 2011). Moreover, counselees/clients can look for services in ways that allow for more prominent anonymity, security, and privacy, thereby improving any potential negative paradigm related to seeing a pastoral/spiritual counselor or psychologist (Barak et al., 2008; Cartreine et al., 2010; McAdams & Wyatt, 2010). Anonymity, intangibility, and more time to structure reactions were imperative to King et al.’s (2006) young subjects. There is less risk of partiality, since age, sex, race, ethnicity, and socio-economic status are frequently more difficult to perceive in a separate environment (Barak et al., 2009; Miller & Gergen, 1998). At the same time, there is much more to do with other people who are geographically different in dealing with a problem (Green-Hamann et al., 2011; Holmes & Foster, 2012). 

Online/cyber counselees/clients frequently converse more unreservedly when they feel they are in a secure, non-judgmental environment and habitually make more profound revelations to the pastoral/spiritual counselor or psychologist, and disclose their problems sooner than they might in a face-to-face session (King et al., 2006; Goss & Anthony, 2004; Reynolds et al., 2006; Suler, 2004). Text-based communication creates a composed record that possibly benefits both counselees/clients and pastoral/spiritual counselors or psychologists (Kessler et al., 2009), and Goss and Anthony (2004) found that counselees/clients had improved control of the treatment session since they are able to hang up, log off, or move away from the camera. In truth, numerous counselees/clients of virtual stages cite the capacity to be in control of their environment as one of the reasons they take part in online/cyber counseling (Leibert et al., 2006; Partala, 2011).

## The Disadvantages of Online/Cyber Counseling

In spite of the advantages of online counseling, pastoral/spiritual counselors or psychologists have frequently been hesitant to make use of technological advances, often with good reason. Whereas pastoral/spiritual counselors or psychologists can at present ignore geographical restrictions, concerns over time-planning, jurisdictional licensure, and restricted awareness of territorial standards and occurrences have surfaced (Green-Hamann et al., 2011; Holmes & Foster, 2012; McAdams & Wyatt, 2010; West & Hanley, 2006). The need for satisfactory preparation, mechanical information, adequate private computer access, and support for the uneducated have also been raised (American Counseling Association (ACA), 2014; McAdams & Wyatt, 2010; Mallen & Vogel, 2005; Miller & Gergen, 1998). In the USA, improvements including the Health Information Portability and Accountability Act (HIPAA) and Health Information Technology for Economic and Clinical Health Act (HITECH) have caused pastoral/spiritual counselors or psychologist to carefully consider the utilization of electronic communication (Kanani & Regehr, 2003).

At long last, the need for non-verbal data ordinarily picked up from watching body language and other visual and verbal prompts is changing the counseling dynamic and has caused a few to address the viability of separate counseling (Barak et al., 2009; Cartreine et al., 2010; Suler, 2004). As an illustration of this, the young people in Bambling et al.’s (2008) and King et al.’s (2006) research accepted that their pastoral/spiritual counselors or psychologists regularly confused the enthusiastic substance of their messages. In addition, Situmorang (2018), Situmorang, Mulawarman, and Wibowo (2018a, 2018b), and Situmorang, Wibowo, and Mulawarman (2018) found that if the counseling process uses music therapy, especially if the counselor and counselee/client play music together online, there will be a sound delay.

## Skills, Characteristics, and Other Considerations for Online/Cyber Counseling

Pastoral/spiritual counselors or psychologists must consider a few variables when committing to online/cyber counseling. In this case, the pastoral/spiritual counselor or psychologist must be able to understand the complex characteristics of each counselee/client (Kanani & Regehr, 2003). At the same time, they must have suitable specialized information and have a certain level of familiarity with errors, miscommunication, and working with different societies (ACA, 2014). Pastoral/spiritual counselors or psychologists ought to screen out potential counselees/clients requiring high levels of contact and those with serious pathology, hazardous behaviors, and troubles with reality testing (Rochlen et al., 2004; Suler, 2001). They must also evaluate the counselee/client's familiarity with technology and their expressive abilities in the online/cyber counseling process. Barnett (2005) and Fenichel et al. (2002) recommended that counselees/clients with high levels of inspiration, excellent reading and writing abilities, and the capacity to recognize and clarify miscommunications may be most appropriate for online/cyber counseling.

Moreover, pastoral/spiritual counselors or psychologists utilizing online/cyber media ought to have the capacity to sort rapidly, compose expressively, and be proficient at online communication, encryption, and Web-browser administration. Analysts have proposed utilizing more unequivocal methods of communication when composing is the most important modality (Allerman, 2002; Barak, Klein, et al., 2009; Suler, 2004), including capitalization, emoticons (utilizing written images such as smiley faces for specific feelings), bold, italic, or colored type, and other methods of complex accentuation. Portraying sentiments and body language may also help towards more viable communication (Abney & Cleborne, 2004). Cook and Doyle (2002) and Wang et al. (2014) found that virtual meeting facilitators needed to be knowledgeable about the stage being utilized and be able to multitask between technical/logistical components and helping aptitudes. The capacity to move rapidly between separate modalities and face-to-face counseling has, moreover, become well known (Dowling & Rickwood, 2013; Fenichel et al., 2002).

## Counseling Themes Particularly Germane to Pastoral/Spiritual Counseling Based on the Impact of the COVID-19 Outbreak on Mental Health

Nowadays, essential significance is given to the physical wellbeing that incorporates treatments and treatment to pneumonic indications. Furthermore, by paying attention to the issue of mental problems that occurred during COVID-19 outbreak resulting from the viral infection, isolation, restricted social activities, disturbed sleep, lockdown, and fake news; culminating in stress, anxiety, and episodes of depressive reactions (Khan et al., 2020; Zarocostas, 2020).

## Online/Cyber Counseling as the Best Option During the COVID-19 Outbreak

For the first time in the world’s history, all cities around the world are experiencing lockdown, marked by the increasing fear displayed by the media during COVID-19 outbreak (Hui et al., 2020; Yang et al., 2020). In addition to an increasing number of deaths, fast transmission and intense grief are increasing mental illness and uneasiness among people. People in this world are under normal conditions which will lead to less productive behavior. Individuals are frightened of being contaminated by their colleagues and family individuals; hence, they are inclined to stay separated and locked down, and to overuse electronic gadgets. For non-natives, the conditions are more extreme as nations have begun repatriating some individuals because of the stress caused by COVID-19 (Holmes & Foster, 2012). In this way, an appropriate framework must be created to teach individuals through online/cyber counseling. In addition, they ought to be given counseling treatments and mental health help where required.

## Implications for Pastoral/Spiritual Counselor or Psychologist Educators and Credentialing Bodies

To be successful in any setting, pastoral/spiritual counselors or psychologists must be prepared, and special conditions require specialized preparation (Jones, 2013). Pastoral/spiritual counselors or psychologist teachers ought to start introducing prospective pastoral/spiritual counselors or psychologists to this new medium in order to extend the knowledge and viability of these future specialists, whilst also focusing on and encouraging specialized preparation (Kehoe, 2016; Lau et al., 2013). While some preparation and credentialing does exist (e.g., the Distance Pastoral/Spiritual Counselor or Psychologist Credential in the US), more ought to be done. There are also number of legitimate and moral issues that are either conflictingly tended to or not tended to at all.

It is additionally vital to recognize that lawful and moral measures change from country to country. Authorizing bodies must seriously consider the impact of the COVID-19 pandemic, so as to create new and open types of services (Green-Hamann et al., 2011). Organizational structures (e.g., proficient organizations and credentialing and permitting bodies) ought to begin to develop ways of addressing modern methods of acquiring proficiency and skills, and further investigation is required to advise on the best practices. 

## Updating the Pastoral/Spiritual Counselor or Psychologist Education Curriculum During the COVID-19 Outbreak

Online/cyber counseling has been around for decades and is not a new phenomenon. In truth, mental health services have been available since 1982 through the utilization of online/cyber self-help groups (Holmes & Foster, 2012; Kanani & Regehr, 2003). The forerunner to online/cyber counseling was established over a decade ago, in 1995, when primary fee-based treatment was set up on the Internet (Mallen & Vogel, 2005; Skinner & Zack, 2004). Although innovation is progressing and new techniques such as video chat are utilized, the concept of online treatment is not new (McCrickard & Butler, 2005). Since this kind of treatment is not a new phenomenon, why are our graduate students not learning about it? Why is this not included within education modules?

The ultimate suggestion for curriculum designers is that there is clearly a need for instructive material within current counseling education courses. In a standard program there is no focus on online/cyber counseling within the dialogs, courses, or materials. This also confirms research findings, since less than 1% of dynamic pastoral/spiritual counselors or psychologists report having had data displayed to them about online/cyber counseling (Abney & Cleborne, 2004; McKenna & Bargh, 2000; Wells et al., 2007). It appears that a clear shortage has been identified, since this more youthful generation of upcoming pastoral/spiritual counselors or psychologists shows itself to be enthusiastic about memorizing more, whereas their educators show themselves to be lagging behind in providing them with the required and wanted instruction, especially during the COVID-19 outbreak.

## Conclusion and Recommendations

The rise and the impact of information and communications technology (ICT) have had a tremendous effect on the field of pastoral/spiritual counseling, particularly Internet innovation. Through the Internet today, especially during the COVID-19 outbreak, everyone who works in the mental health field can provide free online/cyber counseling services by advertising it as a form of humanity towards others. The history of computer and Web utilization in counseling has added another level to the work of pastoral/spiritual counselors or psychologists, especially during the COVID-19 pandemic outbreak. Understanding the history of the advancement and transformation of online/cyber counseling can bring modern information to pastoral/spiritual counselors or psychologists. For the longer-term discourse and investigation, the issues and opportunities of online/cyber counseling services can also be taken care of. This will enhance information about modern treatments, as well as giving the historical context for the increasing use of online/cyber counseling. 

Furthermore, pastoral/spiritual counseling education students right now have had no formal instructive material on this subject in their pastoral/spiritual counselor or psychologist education program. I propose that advance planning should be conducted in order to examine their particular requirements. Pastoral/spiritual counseling program chairpersons, educational programs chiefs, and course engineers ought to note this clear demand for current graduate counseling students to discover information about online/cyber counseling. The current pastoral/spiritual counseling programs need to be upgraded to reflect the changes within the counseling field and the requirements of students.

## Declaration of Conflicting Interests

The author declared no potential conflicts of interest with respect to the research, authorship, and/or publication of this article.

## Funding

The author received no financial support for the research, authorship, and/or publication of this article.
